# Biomarker-indicated extent of oxidation of plant-derived organic carbon (OC) in relation to geomorphology in an arsenic contaminated Holocene aquifer, Cambodia

**DOI:** 10.1038/s41598-017-13354-8

**Published:** 2017-10-12

**Authors:** Daniel Magnone, Laura A. Richards, David A. Polya, Charlotte Bryant, Merren Jones, Bart E. van Dongen

**Affiliations:** 1 School of Earth and Environmental Sciences, The University of Manchester, Williamson Building, Oxford Road, Manchester, M13 9PL United Kingdom; 2 Williamson Research Centre for Molecular Environmental Science, The University of Manchester, Williamson Building, Oxford Road, Manchester, M13 9PL United Kingdom; 3 NERC Radiocarbon Facility, Scottish Enterprise Technology Park, Rankine Avenue, East Kilbride, G75 OQF United Kingdom; 4 Present Address: School of Geography, University of Lincoln, Brayford Pool, Lincoln, Lincolnshire, LN6 7TS United Kingdom

## Abstract

The poisoning of rural populations in South and Southeast Asia due to high groundwater arsenic concentrations is one of the world’s largest ongoing natural disasters. It is important to consider environmental processes related to the release of geogenic arsenic, including geomorphological and organic geochemical processes. Arsenic is released from sediments when iron-oxide minerals, onto which arsenic is adsorbed or incorporated, react with organic carbon (OC) and the OC is oxidised. In this study we build a new geomorphological framework for Kandal Province, a highly studied arsenic affected region of Cambodia, and tie this into wider regional environmental change throughout the Holocene. Analyses shows that the concentration of OC in the sediments is strongly inversely correlated to grainsize. Furthermore, the type of OC is also related to grain size with the clay containing mostly (immature) plant derived OC and sand containing mostly thermally mature derived OC. Finally, analyses indicate that within the plant derived OC relative oxidation is strongly grouped by stratigraphy with the older bound OC more oxidised than younger OC.

## Introduction

Groundwater arsenic concentrations in excess of the World Health Organisation’s provisional guide line value of 10 µg/L^1^ blight much of South and South East Asia^2,3^ and have been linked to devastating health impacts in rural populations^4^. Many of these groundwaters are hosted in the deltas of circum-Himalayan rivers (*i.e*. those rivers which drain from the Himalayas)^2,3,5,6^. It is widely accepted that arsenic is adsorbed to, or incorporated within, iron-(hydr)oxide minerals hosted in the aquifer sediments, and that within shallow, reducing aquifers this arsenic may be released when organic carbon (OC) reacts with these iron-(hydr)oxide phases^7^ in a microbially facilitated reaction^8^.

The high arsenic groundwaters generally exist in shallow (<50 m) Holocene or Pleistocene aquifers^3,9,10^. Connectivity between surface landforms and the facies architecture of the subsurface deposits, may affect the bioavailability of sedimentary OC to the microbial community and thus potentially impact arsenic release^11–21^. For example, sandy windows within clay caps may allow a potential pathway for young surface derived OC to penetrate the aquifer to depth^22,23^. Furthermore, upwelled, thermally mature petroleum from reservoirs beneath the aquifer may also utilise pathways through the sandy layers^11,12,14,16,17^ becoming bioavailable to the microbial community and facilitating arsenic release^12,13^. However, despite the importance of grainsize and stratigraphy to OC bioavailability, to the authors’ knowledge, no studies have used organic geochemical proxies to quantitatively analyse the levels of oxidation of plant derived OC and relate it to the geomorphological and depositional structure and history in these aquifers.

OC is oxidised during microbial degradation^8,13^, thus quantifying oxidation indicates levels of degradation which might be indicative of differences in bioavailability of OC within stratigraphic secessions or other oxidation factors. OC follow a well-known oxidation pathway from alkanes (the most reduced), through alcohols, aldehydes, alkanoic acids and finally to inorganic carbon (the most oxidised)^24^. This pathway can be utilised to produce the *n*-alkanoic acid to *n*-alkane ratio which is an oxidation proxy for materials of similar origin (*i.e*. all plant-derived or all petroleum-derived). Assuming that for samples of similar origin, the ratio of high molecular weight (HMW) *n*-alkanoic acids or alcohols to HMW *n*-alkanes is initially comparable, then the ratio will indicate relative differences in levels of oxidation between samples^25–27^. This proxy is only valid for OC of the same origin as they must start with a similar ratio of HMW *n*-alkanoic acids or alcohols to HMW *n*-alkanes. To test whether samples are of similar origin, the thermal maturity of HMW *n*-alkanes is used to indicate if samples are thermally mature (petroleum-derived) or immature (plant-derived). This is measured using the ratio of odd to even chained HMW hydrocarbons in a proxy called the carbon preference index (CPI)^28,29^. Additionally the bulk proxy, the C/N ratio can be used to distinguish the origin of OC^30^.

The main aims of this study are to 1) establish the depositional history of the stratigraphy of the study site within the context of regional Holocene environmental change; 2) understand how the distribution and source of OC varies with the distribution of grain size within the stratigraphy; 3) assess the relative oxidation of OC within the stratigraphic framework.

### Study site and Geological Setting

**Figure 1:**
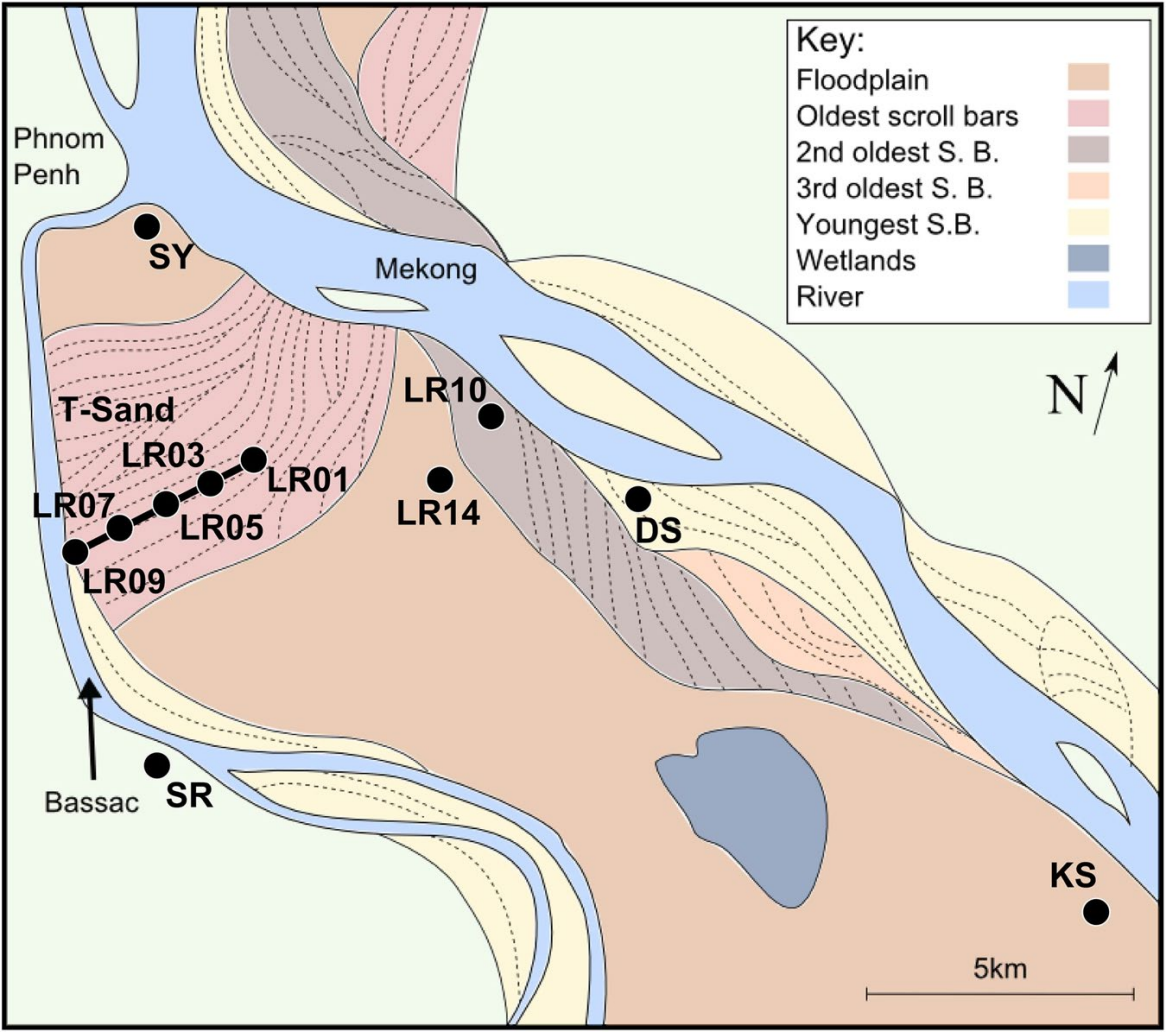
Schematic sketch showing geomorphological cut crossing relationships of north-eastern Kandal Province, Cambodia, based on data by Papacostas *et al*.^44^. Also shown are the locations of study sites LR01, LR03, LR05, LR07, LR09, LR10, LR14 (this study and^36^), SY^12^, KS^38^, DS and SR^14^. Note that wetlands area is approximate and size changes seasonally. This figure was produced using Inkscape 0.91 (https://inkscape.org/en/download/windows/).

The study site is located in Kandal Province, Cambodia, a highly studied region with widespread high groundwater arsenic concentrations^5,9,10,22,23,31–35^. Groundwater arsenic concentrations and other groundwater chemical parameters for this study site are presented by a related study^36^. Kandal Province is located in the lowlands of Cambodia south of Phnom Penh with the study site between the Mekong River and its distributary the Bassac River (Fig. 1). The lowlands of Cambodia are dominated by the Mekong River system, which is one of the great Asian rivers draining from the Himalayas^37^. The sediments in this region are mostly Holocene to a depth of at least 30 m. The early history (12,000 to 7,000 years BP) is dominated by tidal processes due to rising sea-levels during this period^38^ which affected much of the Mekong delta^39,40^. At about 7,000 years BP sea-level rise ceased and has been falling slowly since at least 4,000 years BP. Over the last 6,000 to 7,000 years the delta has prograded into the South China Sea due to the high sedimentation rate of the Mekong river system^38–41^.

Many of the circum-Himalayan rivers are dominated by monsoon processes where the monsoon rain causes greater and increased river water levels and thus frequent flooding. However in the Mekong of the Cambodian lowlands whilst severe flooding still occurs regularly it is less pronounced due to the effects of Tonle Sap, a large fluvial lake in the centre of Cambodia, which is connected to the Mekong by the Tonle Sap River. During the rainy season water flow is in a north westerly direction from the Mekong River into the Tonle Sap with water accumulating in the lake, during the dry season flow reverses flowing south east into the Mekong from the lake. This flow regime means that the seasonal difference in water levels in the Mekong is considerably less than in other circum-Himalayan rivers. This feature results in fewer flooding events and has an important effect on sedimentation downstream of Tonle Sap^42^. The onset of this seasonal reversal in flow at Tonle Sap, referred to as the flood-pulse, occurred between 4,000 and 3,600 ^14^C years BP due to a change in monsoon intensity; the modern system appears to have started 1,660 ^14^C years BP^43^.

Within the study area, Landsat images and interpretation of sedimentary cross-cutting relationships show that at least four generations of scroll bars exist^44^. The oldest of these is perpendicular to the present day Mekong whilst more recent scroll bars run parallel to the modern Mekong and are probably active features (Fig. 1). The oldest sediments are most likely the floodplain deposits into which subsequent river channels have eroded and deposited sediment.

Representative sites with contrasting geomorphological features were selected for study^36^. In this manuscript the nomenclature “LRXX-##” is used; LRXX refers to the site location and ## refers to the sample depth in metres. The sites from LR01 to LR09 make up the transect T-Sand^36,45^, of which key sites were selected for the analysis reported in this manuscript. T-Sand is a 4.5 km 2D transect running along the oldest generation of scroll bars from the central wetlands to the Bassac river. A pre-sampling electrical resistivity tomography here indicated it was probably a largely layer cake structure consisting of sands and clays^36,46,47^. Previous analyses on single boreholes indicate that this structure is associated with distinctive OC composition distribution^14,16,17^. Site LR14 was selected to provide a sample representative of the flood plain. LR10 was selected to provide a sample representative of younger scroll bars than on T-Sand (Fig. 1). Additional data from other sites (SY, DS, SR and KS) conducted earlier in the same region using similar methods have also been used to give a wider regional setting to this study^12,14,38,41^.

## Results

### Grainsize distribution, TOC and age of sediments

**Figure 2:**
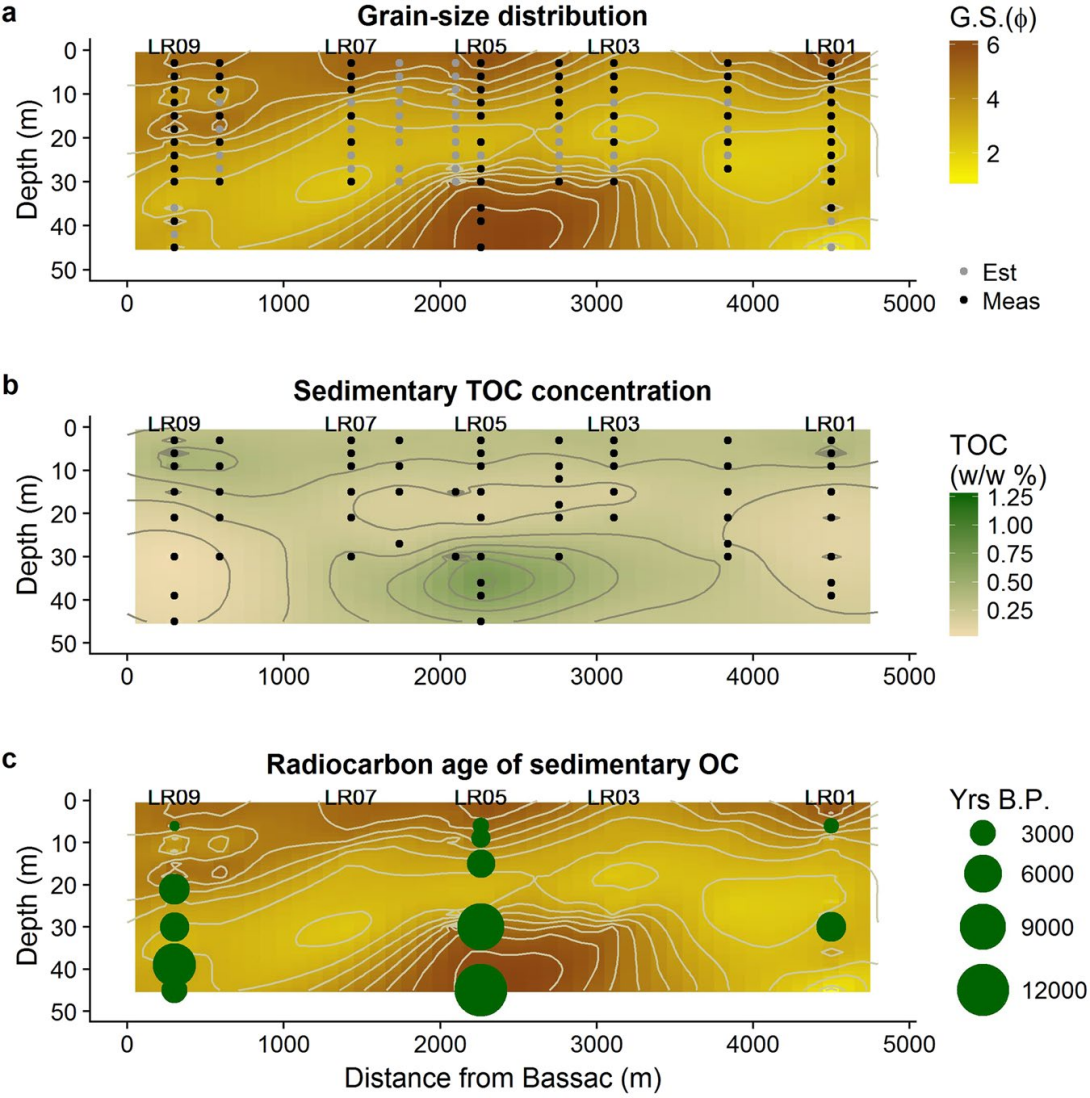
(**a**) Kriged results for the distribution of sedimentary Folk and Ward mean grain size (φ = −log2 (D/D_0_), where D is the diameter of the sample in mm and D_0_ is the reference constant 1 mm), (**b**) TOC % (w/w), and (**c**) distribution of radiocarbon dates on T-Sand of this study (Fig. 1). Black dots are measured values from which kriging was calculated grey dots are estimated values used for co-kriging. Note that the separate profiles of LR10 and LR14 are not shown.

The Folk and Ward based mean grain size of the sediments (see supplementary information Table 1) ranges from 1.07 mm for the very coarse sand at LR02-27, to 0.005 mm for the fine silt at LR05-45 (Fig. 2). On T-Sand there are three layers; on the top is a silty and clayey cap, a middle sandy layer and a deep silt wedge. The deep silt wedge has grainsizes <0.031 mm (LR05 to LR03, 30–45 m) and the middle sandy sediments are >0.063 mm (Fig. 2). The clay cap extends across the top of the entire transect, with the exception of a sandy window at LR03. At LR09 and LR08 the clay extends to about 21 to 18 m, however across most of T-Sand it is much shallower and it does not extend far below 9 m (Fig. 2). LR10 is predominantly coarse silt and fine sand with grain sizes ranging from 0.038 to 0.25 mm, whereas LR14 is silty, with grain sizes ranging from 0.006 to 0.058 mm.

**Figure 3:**
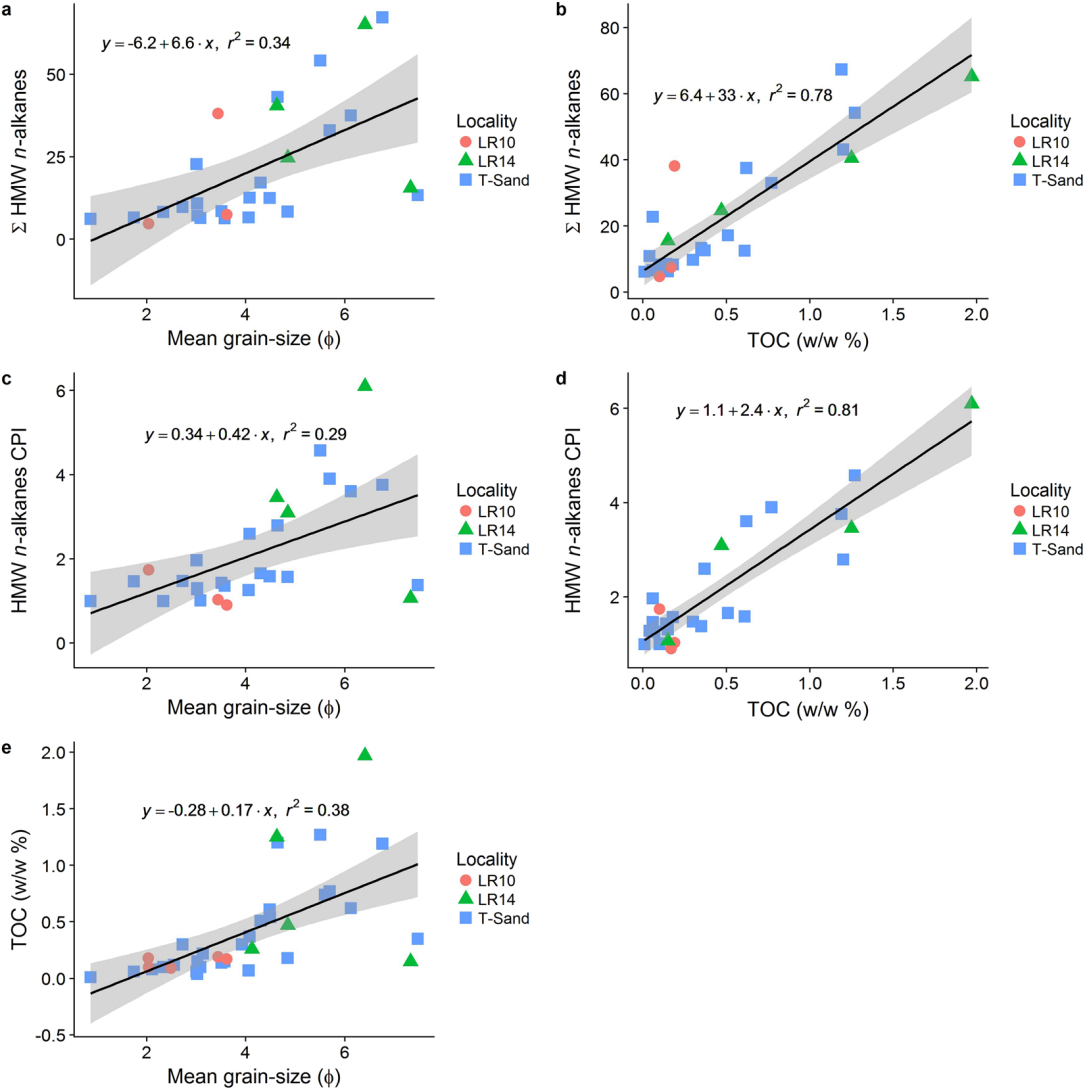
The correlations between ΣHMW *n*-alkanes concentration (ng/g Sed) vs. (**a**) Mean grain-size (φ); (**b**) TOC (w/w %); HMW *n*-alkanes CPI vs. (**c**) Mean grain-size (φ); (**d**) TOC (w/w %); and (**e**) TOC (w/w %) vs mean grain-size (φ). All plots show linear regression line and one standard error in grey for all data. (φ = −log2 (D/D_0_), where D is the diameter of the sample in mm and D_0_ is the reference constant 1 mm).

TOC concentrations in these sediments (supplementary information Table 1) are weakly associated with the sedimentary grain size distribution (R^2^ = 0.38, p-value = 5 × 10^−5^, Figs 2 and 3). The TOC concentrations in the deep silty wedge range from 0.7 to 1% (w/w), in the middle sandy layers the concentration is lowest, ranging from <0.05% (w/w) at multiple locations, to 1.0% (w/w) at LR02-30, whilst the clay cap has a medium high TOC content with the lowest values from 0.5 and the highest at 1.2% (w/w) at LR01-6 and LR09-6 (Fig. 2). Total nitrogen (TN) concentrations range from below detection limit to 0.26% (w/w) and correlate positively with TOC (R^2^ = 0.61, p-value = 5 × 10^−13^, supplementary information Table 1). The C/N ratio ranges from 1.51 to 16.5 (supplementary information Table 1).

The radiocarbon age of the OC ranges from 1,500 ^14^C years BP (83 pmc; LR09-6) to 12,100 ^14^C years BP (22 pmc; LR05-45), generally sites age with depth. On T-Sand the oldest sediments are in the deep silty wedge with ages >9,000 ^14^C years while the youngest sediments are in the shallow clay cap deposits with ages <1,500 ^14^C years with intermediate ages for the middle sandy sediments. The shallow samples from the LR10 and LR14 are significantly older than those on T-Sand with ages of 2,600 ^14^C years BP (72 pmc) at LR10-6 and 6,400 ^14^C years BP (45 pmc) at LR14-6. The deeper sediments at 30 m at LR14 are the same age as the deep silty wedge on T-Sand 9,500 ^14^C years BP (31 pmc). The δ^13^C of OC ranges from −32.3 to −25.2‰ with a mean of −27.2‰ on the data measured by this study, significantly higher values exist at a handful of sites from the earlier studies with values of −22.0, −23.0 and −23.7‰ at KS-28.1, DS-60 and DS-70 (Table 1, Fig. 2).

**Table 1:** Sedimentary Radiocarbon Data, Kandal Province, Cambodia. See Fig. 1 for the location of samples.

Sample^a^	TOC δ^13^C‰_VPDB_ ± 0.1	Conventional Radiocarbon Age		Calibrated age range (Years BP)		Publication code	Reference^b^
		^14^C % absolute modern ± 1σ	^14^C Years BP ± 1σ	From-to	Probability		
LR01-6	−25.9	81.42 ± 0.38	1588 ± 37	1555–1396	0.954	SUERC-64718	TS
LR01-30	−25.4	62.56 ± 0.28	3705 ± 36	4150–3965	0.927	SUERC-64719	TS
LR05-6	−25.2	80.46 ± 0.66	1683 ± 35	1695–1522	0.948	SUERC-67120	TS
LR05-9	−25.5	78.09 ± 0.34	1923 ± 35	1986–1815	0.954	SUERC-67124	TS
LR05-15	−25.9	65.27 ± 0.29	3363 ± 35	3696–3550	0.887	SUERC-67125	TS
LR05-30	−27.9	30.66 ± 0.15	9432 ± 40	10794–10498	0.930	SUERC-64126	TS
LR05-45	−26	22.24 ± 0.13	12011 ± 45	14031–13746	0.954	SUERC-67127	TS
LR09-6	−25.7	83.03 ± 0.36	1430 ± 35	1385–1290	0.954	SUERC-67128	TS
LR09-21	−27.8	60.96 ± 0.27	3911 ± 35	4431–4239	0.954	SUERC-67129	TS
LR09-30	−27.8	63.4 ± 0.28	3597 ± 35	3987–3828	0.940	SUERC-67130	TS
LR09-39	−32.3	36.64 ± 0.18	8002 ± 40	9010–8719	0.954	SUERC-67134	TS
LR09-45	−30.8	68.89 ± 0.30	2930 ± 35	3175–2963	0.954	SUERC-67135	TS
LR10-6	−27.2	72.17 ± 0.32	2555 ± 35	2754–2496	0.954	SUERC-67136	TS
LR10-27	−27.4	65.03 ± 0.30	3393 ± 37	3724–3560	0.933	SUERC-67137	TS
LR14-6	−26.2	45.31 ± 0.22	6295 ± 39	7309–7161	0.954	SUERC-67138	TS
LR14-30	−28.6	30.59 ± 0.16	9450 ± 41	10787–10571	0.930	SUERC-67139	TS
KS-4.07	−26.7	n.r.	700 ± 40	701–558	0.954	Beta-192747	T07
KS-7.08	−27.9	n.r.	6250 ± 40	7265–7019	0.954	Beta-192748	T07
KS-7.9	−27.9	n.r.	6620 ± 40	7570–7440	0.954	Beta-192749	T07
KS-8.33	−29.1	n.r.	6470 ± 40	7458–7293	0.954	Beta-192750	T07
KS-9.08	−28.1	n.r.	7130 ± 40	8020–7922	0.832	Beta-192751	T07
KS-9.6	−30.3	n.r.	7030 ± 40	7954–7786	0.946	Beta-192752	T07
KS-10.5	−28.6	n.r.	7150 ± 40	8031–7926	0.911	Beta-192753	T07
KS-12.3	−30.0	n.r.	6550 ± 40	7520–7417	0.884	Beta-192754	T07
KS-12.7	−25.7	n.r.	6760 ± 40	7675–7570	0.954	Beta-192755	T07
KS-28.1	−22.0	n.r.	8180 ± 40	9262–9020	0.954	Beta-192756	T07
SR-13	−27.1	n.r.	7759 ± 51	8627–8425	0.954	SUERC-9245	vD08
SR-19	−27.5	n.r.	7732 ± 51	8595–8417	0.954	SUERC-9246	vD08
DS-0	−28.1	n.r.	6216 ± 44	7250–7004	0.954	SUERC-9235	vD08
DS-15	−27.0	n.r.	8177 ± 54	9282–9009	0.954	SUERC-9236	vD08
DS-23	−26.6	n.r.	7930 ± 52	8984–8610	0.954	SUERC-9237	vD08
DS-27	−25.2	n.r.	9040 ± 61	10300–10119	0.813	SUERC-9239	vD08
DS-40	−25.5	n.r.	5370 ± 41	6280–6005	0.954	SUERC-9242	vD08
DS-54	−25.8	n.r.	5293 ± 41	6190–5984	0.896	SUERC-9243	vD08
DS-60	−23.0	n.r.	8241 ± 41	9323–9085	0.864	SUERC-9244	vD08
DS-70	−23.7	n.r.	4937 ± 41	5743–5594	0.954	SUERC-9565	vD08
SY-9	−25.1	n.r.	1532 ± 31	1523–1353	0.954	SUERC-9228	R07
SY-28	−25.5	n.r.	4218 ± 38	4855–4625	0.954	SUERC-9232	R07

^a^Samples from this study labelled LRXX-## where LRXX refers to the location (see Fig. 1) and ## refers to the depth in metres. Also, samples from earlier studies labelled $$-## where $$ is the location (see Fig. 1) and ## is depth in metres. n.r. value not reported by original study.

^b^References with the following code: TS = This study, T07 = Tamura *et al*.^38^, vD08 = van Dongen *et al*.^14^ and R07 = Rowland *et al*.^12^.

### Lipid analysis

**Figure 4:**
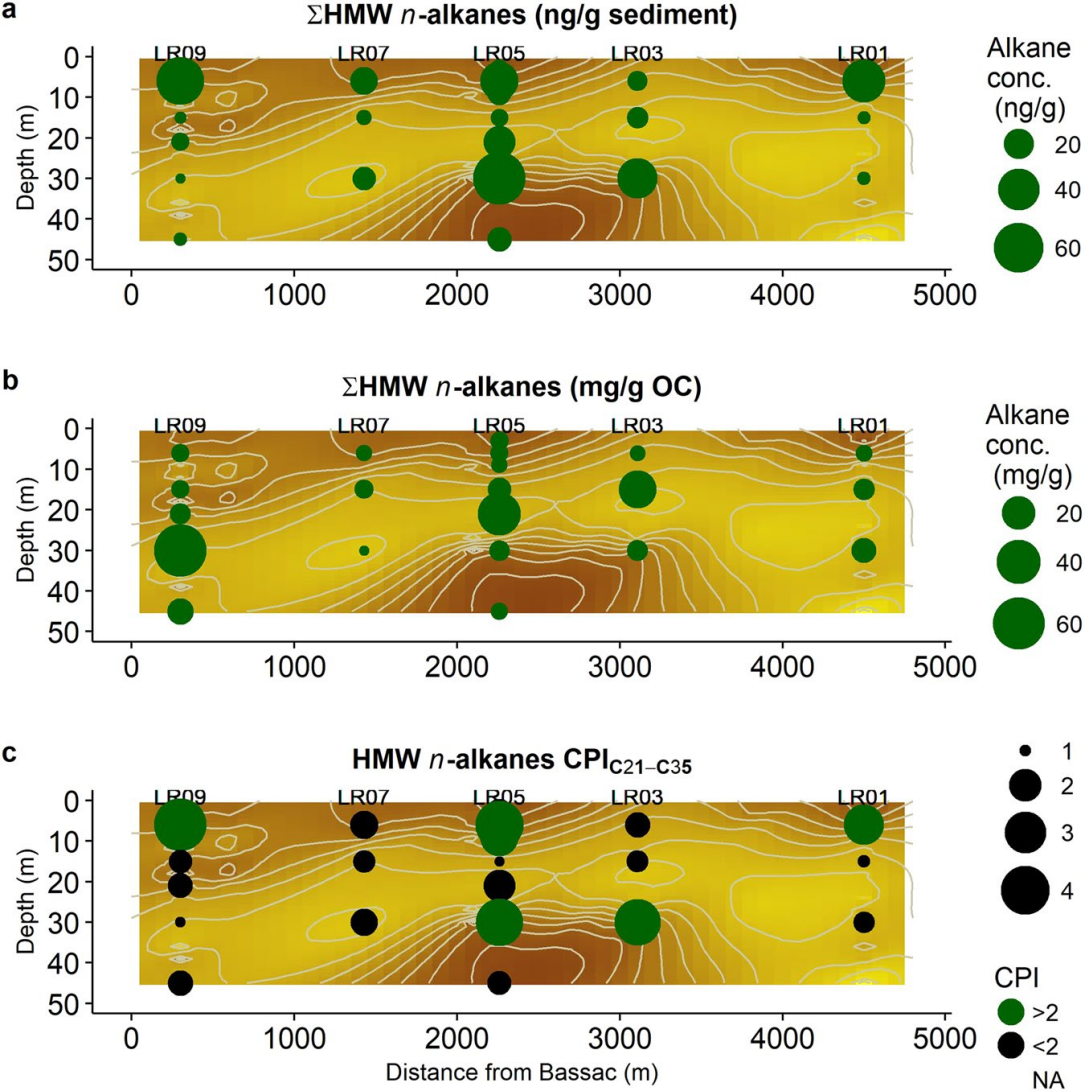
Distribution of (**a**) HMW *n*-alkane (Σ(C_21–35_)) concentration per gram sediment, (**b**) HMW *n*-alkane concentration per gram OC and (**c**) HMW *n*-alkane CPI along T-Sand (Fig. 1) plotted over grainsize (Fig. 2).

Lipid analyses of the sediments indicates the presence of low but quantifiable amounts of HMW *n*-alkanes with concentrations ranging from 6 to 67 ng/g sediment (Table 2) and a distribution that mostly follows the TOC concentration (R^2^ = 0.78, p-value 5 × 10^−11^, Fig. 3) with highest concentrations predominantly in the clay layers (Table 2, Fig. 4, representative chromatograms are presented in supplementary information). The distribution of HMW *n*-alkanes as a proportion of OC ranges from 2 to 62 mg/g OC, with the highest proportions found in the sandy layers and the lowest in the clay sediments (Table 2). Those with a CPI_21–35_ > 2 are mostly restricted to the clay layers (both the clay wedge and the clay cap) with a notable exceptions of LR05-21 which is a sandy sediment with a relatively high CPI_21–35_ of 2 and LR05-45, which is in the clay wedge but has a relatively low CPI of 1.4 (Table 2, Fig. 4). Note that LR03-6, a shallow sediment in the sandy window, has a relatively low CPI of 1.5 (Table 2, Fig. 4). CPI_21–35_ of HMW *n*-alkanes has a very strong correlation with TOC (R^2^ = 0.81, p-value 6 × 10^−11^), indicating that the more thermally immature the OC the more OC is present (Fig. 3). The HMW *n*-alkanoic acids have a similar magnitude of concentration to the HMW *n-*alkanes, ranging from 0.18 ng/g sediment at LR03-6 to 202 ng/g of sediment at LR05-30 or 0.06 mg/g of OC at LR03-6 to 25.4 mg/g of OC at LR05-21 (Table 2). The CPI_22–34_ is consistently high with a range from 3.26 at LR05-45 to 9.96 at LR01-6. The *n*-alkanoic acid to *n*-alkane ratio ranges from 0.06 to 0.75. LR14-15 is notable for having an exceptionally high HMW *n-*alkanoic acid concentration of 914 ng/g sediment. Other than this sample the HMW *n-*alkanoic concentration on T-Sand and at LR10 and LR14 were similar, with respective mean concentrations of 19 ng/g (n = 20) and 18 ng/g sediment (n = 6). Finally, at LR14-15 the *n*-alkanoic acid to *n*-alkane ratio is the highest ratio reported by this study with a value of 0.96.

**Table 2:** Total lipid analysis of sedimentary OC for Kandal Province, Cambodia.

Sample^a^	HMW *n*-alkanes (C_21–35_)			HMW *n*-alkanoic acids (C_20–30_)			Acid/alkane Ratio^e^	Reference^f^
	Conc Sed. (ng/g)^b^	PropOC (mg/g)^c^	CPI_21–35_^d^	Conc Sed. (ng/g)^b^	PropOC (mg/g)^c^	CPI_20–30_^d^		
LR01-6	43	3.6	2.8	39	3.27	10.0	0.48	TS
LR01-15	6.4	6.2	1.0	0.3	0.27	4.8	N/A	TS
LR01-30	6.6	9.6	1.3	1.8	2.63	3.3	N/A	TS
LR03-6	9.8	3.2	1.5	0.2	0.06	5.3	N/A	TS
LR03-15	11	26	1.3	2.3	5.64	5.3	N/A	TS
LR03-30	38	6.1	3.6	46	7.53	7.3	0.55	TS
LR05-3	8.3	4.5	1.6	3.8	2.06	3.0	N/A	TS
LR05-6	33	4.3	3.9	5.8	0.76	6.8	0.15	TS
LR05-9	13	3.4	2.6	0.8	0.20	5.0	0.06	TS
LR05-15	8.3	8.4	1.0	0.7	0.74	4.2	N/A	TS
LR05-21	23	36	2.0	16	25.4	5.9	0.41	TS
LR05-30	67	5.6	3.8	202	16.9	6.2	0.75	TS
LR05-45	13	3.8	1.4	1.1	0.32	3.3	N/A	TS
LR07-6	17	3.3	1.7	2.6	0.50	5.2	N/A	TS
LR07-15	7.2	4.8	1.3	11	7.34	6.6	N/A	TS
LR07-30	13	2.0	1.6	2.1	0.34	5.6	N/A	TS
LR09-6	54	4.3	4.6	66	5.15	6.5	0.55	TS
LR09-15	6.3	4.1	1.4	0.5	0.30	7.9	N/A	TS
LR09-21	8.5	6.0	1.4	1.1	0.76	5.9	N/A	TS
LR09-30	6.1	77	1.0	0.9	11.9	6.6	N/A	TS
LR09-45	6.6	10	1.5	0.3	0.45	4.8	N/A	TS
LR10-6	7.5	4.3	0.9	0.2	0.13	3.2	N/A	TS
LR10-9	38	20	1.0	17	8.97	2.9	N/A	TS
LR10-15	n.d.	n.d.	n.d.	2.4	1.31	5.0	N/A	TS
LR10-30	4.7	4.5	1.7	0.0	0.00	1.1	N/A	TS
LR14-6	16	10	1.1	1.2	0.79	2.5	N/A	TS
LR14-9	65	3.3	6.1	58	2.92	8.4	0.47	TS
LR14-15	40	3.2	3.5	914	73.4	12.6	0.96	TS
LR14-30	25	5.3	3.2	50	10.7	7.9	0.67	TS
SR-13	760	77	3.0	n.d.	n.d.	n.d.	n.d.	vD08
SR-19	1100	89	4.4	n.d.	n.d.	n.d.	n.d.	vD08
SR-25	74	82	2.5	n.d.	n.d.	n.d.	n.d.	vD08
SR-35	81	405	1.3	n.d.	n.d.	n.d.	n.d.	vD08
SR-51	100	200	1.3	n.d.	n.d.	n.d.	n.d.	vD08
DS-10	2100	17	4.0	n.d.	n.d.	n.d.	n.d.	vD08
DS-15	870	113	3.7	n.d.	n.d.	n.d.	n.d.	vD08
DS-23	140	350	1.8	n.d.	n.d.	n.d.	n.d.	vD08
DS-27	74	370	1.1	n.d.	n.d.	n.d.	n.d.	vD08
DS-40	51	510	1.2	n.d.	n.d.	n.d.	n.d.	vD08
DS-54	190	475	1.2	n.d.	n.d.	n.d.	n.d.	vD08
DS-60	78	78	0.9	n.d.	n.d.	n.d.	n.d.	vD08
SY-9	3059	478	7.0	1583	247	3.6	0.34	HR07
SY-28	419	599	2.3	620	886	3.0	0.60	HR07

BDL Below detection limit, n.d. no data, N/A not applicable due to mature CPI.

^a^Samples from this study labelled LRXX-## where LRXX refers to the location (see Fig. 1) and ## refers to the depth in metres. Also, samples from earlier studies labeled $$-## where $$ is the location (see Fig. 1) and ## is depth in metres.

^b^Concentration of aforementioned lipid per mass sediment.

^c^Proportion of aforementioned lipid per mass organic carbon.

^d^Carbon preference index of aforementioned lipid.

^e^HMW *n*-alkanoic acid to Σ(HMW *n*-alkane ratio + HMW *n*-alkanoic acid).

^f^References for data with the following codes: TS = This Study, vD08 = van Dongen *et al*.^14^, HR07 = Rowland *et al*.^12^.

## Discussion

The grainsizes and radiocarbon ages indicate that there are three key ages of deposition. The Early Holocene was dominated by the deposition of clay sediments from about 12,000 years to 6,000 years BP (the early Holocene facies; EHF), followed by sandy deposits from 4000 years to 2000 years BP (mid Holocene facies; MHF) and finally a recent depositional period continuous for the last 2000 years (young Holocene Facies;YHF). The EHF is found at three localities which are the entire depth of LR14, LR05-30 and 45 m on T-Sand and KS below 5 m. The EHF samples at 30 m on T-Sand and LR14 have very similar radiocarbon ages, 9,500 ± 40 ^14^C years BP, however at LR14 the shallow (6 m) sediments are 6,360 ± 40 ^14^C years BP which is considerably older than anything on T-Sand at the same depth (1,700 ± 40 ^14^C years BP; Table 1). The similarity in ages between the deep sediments at LR05 and the age of the sediments at LR14 is strong evidence in favour of the presence of an EHF. Provided that little erosion has taken place at LR14 the 6 m sample represents the latter stages of deposition of the EHF, at around 7,310 to 7,160 cal years BP (equivalent to 6,360 ± 40 ^14^C years BP), which means that sedimentation of EHF ceased with the cessation of sea-level rise in the area at about 7,000 cal years BP^38,41^.

**Figure 5:**
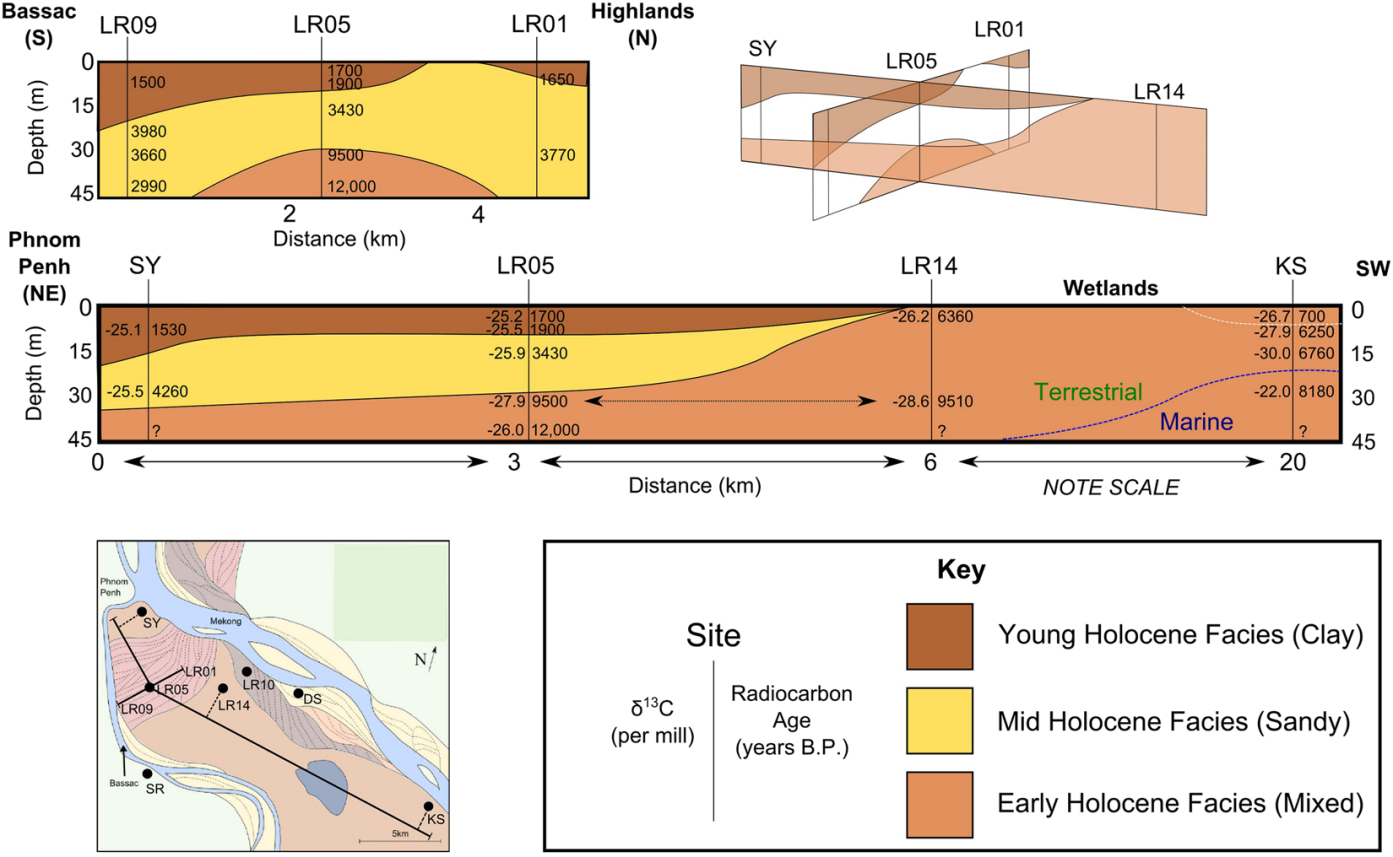
Sketch of cross sections along and perpendicular to T-Sand based on multiple profiles (SY^12^, LR01, LR05, LR09, LR10, LR14 (this study), KS^38^, DS and SR^14^). Sketch of a 3D diagram showing how cross sections relate. Note vertical exaggeration. This figure was produced using Inkscape 0.91 (https://inkscape.org/en/download/windows/).

At KS sedimentary structures indicate that sediments from 12 m to 30 m, deposited during the early Holocene, were tidal deposits^38,41^ and the carbon isotopic values are also indicative of marine deposition^30^. Here the early Holocene deposition also ceased with the cessation of sea-level rise at around 7265 to 7019 cal years BP (6,250 ± 40 ^14^C years BP, 7 m). However, at KS there was a transition from the marine to more terrestrial deposition at about 7675 to 7570 cal years BP (6,760 ± 40 ^14^C years BP, 12 m) which is recorded in both sedimentary structures and the carbon isotopes^38,41^. The carbon isotopes at LR14 and all other sites sampled by this study indicate that they are entirely terrestrial deposits^30^ suggesting that the early Holocene shoreline was located between LR14 and KS (Fig. 5).

The MHF ranges from 4,260 ± 40 ^14^C years BP to 2,990 ± 40 ^14^C years BP (Fig. 5). The contacts between the EHF and the MHF represent unconformities with the appearance of an incision channel at about to 4,855 to 4,625 cal years BP (4,220 ± 40 ^14^C years BP, based on the date from SY-27). The occurrence of this high energy system is concurrent with a major change in monsoon patterns^42^, given an increase in precipitation would increase the load carrying capacity of the Mekong then it is possible that the sandy facies is linked to the change in monsoon, however it could also be a localised geomorphological feature.

The final depositional sequence on T-Sand (and SY) was the shallow clay cap of the YHF. The YHF is not present at LR10 or LR14 or at the high point on T-Sand (LR03) where there is a sandy window through the clay. Given that the YHF is associated with recent flooding events^38,41^, this means that modern flooding events do not affect all areas, LR03 is in raised topography and thus is less susceptible to flooding events.

There are two explanations for the stratigraphy present, firstly, that the deposition was autocyclic and the stratigraphy is a result of avulsion of the major river channel. However, secondly, and perhaps more interestingly, deposition of the YHF started at about 1990 to 1820 cal years BP (1,900 ± 40 ^14^C years BP). These dates coincide with the onset of the modern flood pulse at Tonle Sap which occurred about 1,660 cal years BP and initiated a lower energy period of the Mekong river through the Cambodian lowlands^43^. This would have reduced the load bearing capacity of the river, reducing the possible transportable grainsize. So it is possible that the appearance of the YHF at this study site and the onset of Tonle Sap flood-pulse are connected. However, without a more detailed sedimentary study, it is impossible to conclusively state at this moment which hypothesis is most accurate.

Previous studies have noted the unusual age-depth pattern of some sediments, in that sediments do not always increase in age with depth^14^. At DS sediment age increases with depth down to 27 m but is followed by younger sandy sediments present below 27 m^14^. Similarly, LR09 (this study) has an age of 8,000 ± 40 ^14^C years BP at 39 m depth but at 45 m the age is only 2,930 ± 40 ^14^C years BP. It is notable that this occurs at both DS and LR09 which are near rivers and also in the sandy sediments below 30 m.

Such an age-depth profile is not consistent with any model of sedimentation and is therefore likely to be the result of other factors. There are two potential causes of the unusual age-depth profile. Firstly, the unusual age-depth profiles are in deeper (>35 m) sandy sediments near rivers leading to the possibility of infiltration of river POC into the sedimentary OC. Secondly, the possibility of small levels of contamination cannot be ruled out, sandy sediments are less well consolidated than clays and difficult to sample at greater depths. Given that the sedimentary OC concentrations at these sites are extremely low, low levels of infiltration or contamination could have a relatively large effect on the radiocarbon dates. Thus, caution should be given when interpreting data from sandy sediments below 30 m particularly when a site is located near a river and sampling has involved wet drilling.

The distribution of OC in the sediments in terms of quantity and type is clearly strongly related to grainsize. The TOC correlates moderately with grainsize, with the highest concentrations in the clay sediments (mostly YHF and EHF). Rapid sedimentation has been associated with the accumulation and preservation of OC in this region^44^ and clayey sediments are associated with lower energy clay deposition than higher energy sandy deposition^48^. Given that most POC in the Mekong is clay (<63 μm)^49^ it is expected that most is deposited with clay particles in low energy floodplains. There is a small statistical difference between the concentrations of OC in the YHF and EHF with mean (±standard deviation) concentrations of 0.46 ± 0.28% (n = 23) and 0.78 ± 0.58% (n = 10) respectively and a Student’s p-value of 0.06. This means that to all intents and purposes a similar amount of OC should be considered within both clay facies.

The distribution of different types of organic material is consistent with earlier studies^11,12,14,16,17^, showing that thermally mature HMW *n*-alkanes in these aquifers are largely restricted to the sandy layers: this is probably because these are upwardly migrating petroleum and the sand, unlike the clay, has large pore sizes that can be exploited. The correlation between grainsize and CPI shows that sandy sediments primarily contain thermally mature OC which previous studies have shown to be bioavailable but at relatively low concentrations^12–14,16,17,50^. The results shown in Fig. 4 clearly indicate that the plant material is mostly, but not exclusively, associated with clay layers and that the concentration of plant OC is significantly higher than thermally mature OC. This conclusion, that plant derived OC is mostly restricted to the clay layers, is also supported by another proxy, the C/N ratio. Higher C/N ratios indicate that OC is likely to be plant derived^30^ and at our site clay layers have higher C/N (see supplementary information Table 1), this is consistent with a study of broadly similar sediments in Vietnam^16,19^.

**Figure 6:**
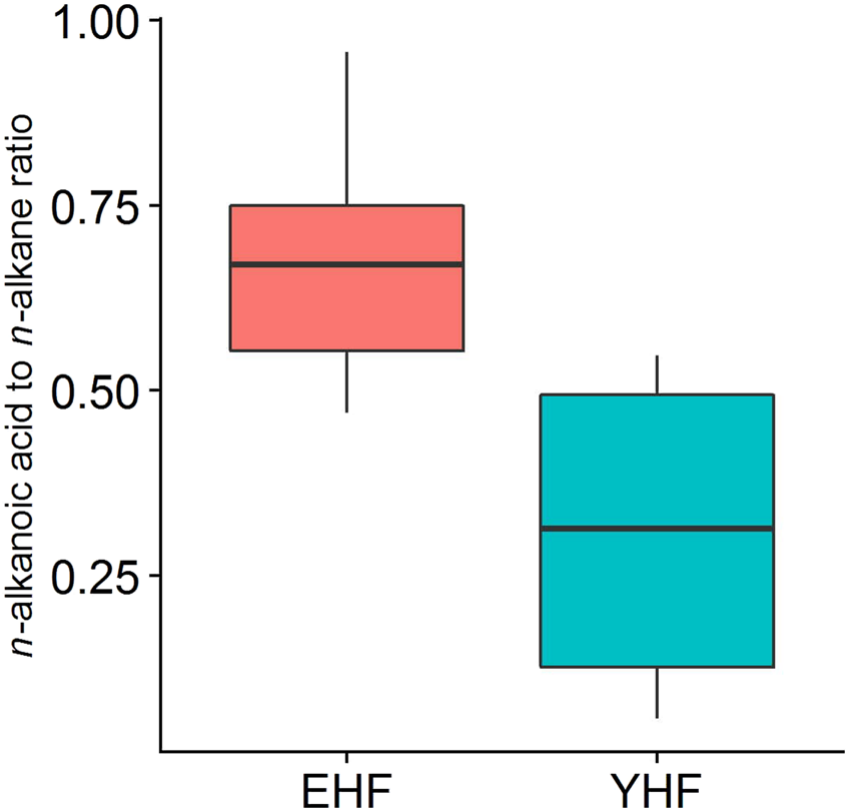
The degradation proxy, *n*-alkanoic acid to *n*-alkane ratio, from the thermally immature samples from Kandal Province, Cambodia, grouped by facies (Early Holocene Facies = EHF, Young Holocene Facies = YHF).

There appears to be a relationship between stratigraphic position of OC and the relative levels of oxidation. The *n*-alkanoic acid to *n*-alkane ratio clearly shows that of the plant OC, that in the EHF has undergone more oxidation than the younger facies (Fig. 6). The levels of oxidation in the EHF are statistically higher than in the YHF (Student’s p-value = 0.02) implying that the older plant OC is more degraded than younger OC, this could be due to the longer time available for degradation in the older layers or could be related to differences in bioavailability between the two layers.

These results demonstrate that geomorphological processes which formed deposits at this study site are quite different to those which formed the deposits at the highly detailed core of KS which is located 20 km further south^38,41^. This is important because many studies of arsenic in groundwater at this site have used KS to interpret these sediments due to the similarities in stratigraphy across much of the Kandal Province area and at KS^20,22,23,35,45,51–56^. This means that, despite its quality, we suggest that KS is not a suitable framework for this area of Kandal Province as the site is under a different geomorphological system. Therefore these results indicate that future studies of this area should use a locally appropriate geomorphological framework for reference in future research.

These results clearly show that the concentrations and bioavailability of sedimentary OC at our study are related to stratigraphy and grainsize which in turn is controlled by wider regional environmental and climatic change throughout the Holocene. Previous studies have already shown that thermally mature (petroleum-derived) OC exists in the sands and is bioavailable, yet the concentrations are very low^11–14,16,17,50^. These results show that a much greater reservoir of plant derived OC exists in the clays both from the early Holocene (>7,000 years BP) and more recent Holocene (<2,000 years BP). The oxidation proxies show that the old plant derived OC is much more oxidised than young plant derived OC. This could be because the older sediments have had more time to oxidise than the younger sediments but it could also relate to differences in the bioavailability between the two layers. If the latter is true then this indicates that variations between sediments and/or the nature of the OC within them could have an effect on the bioavailability of the OC. Understanding what, if any, effect this has had on the release of arsenic is the next important challenge.

## Methodology

### Sediment collection

Coring was performed by manual rotary drilling using a cutting auger attached to the end of a hollow steel pipe up to a maximum depth of 30 or 45 m. For detailed descriptions of the sampling methods see Richards *et al*.^45^. About 100 g (though sample size varied greatly) of sediments was collected every 3 m through manual hammering of a custom made stainless steel sampler attached to a thinner drilling rod which was lowered through the middle of the hollow drilling pipe. Sediment cores were captured in an internal acrylic sampling tube (25 mm or 50 mm) capped with a one-way valve made from bottled water caps and removed from the acrylic sampling tube using a purpose built stainless steel sample plunger or manual hammering.

All sediment samples for organic and inorganic analysis were placed in foil bags which had previously been furnaced to 350 °C for 3 hours to remove any organic contaminants. The foil bags were placed in resealable polyethylene bags and flushed with nitrogen to minimise oxidation. A subset of sediment sample for later grain size analysis was stored in polythene bags (without a foil bag) and not flushed with nitrogen. All samples were placed in a polystyrene chiller containing ice packs before transport within a few hours of collection to the local laboratory where they were re-flushed with nitrogen and frozen. Samples were transported back to Manchester by non-temperature controlled air freight and once in Manchester samples were stored in a freezer at −20 °C, prior to analysis.

### Inorganic and bulk sediment characterisation

Full method details for all techniques are presented in the supplementary information. Total OC (TOC), reported as % w/w relative to total sediment, was measured in the Faculty of Life Sciences, University of Manchester, using an elemental analyser (Vario EL Cube, Elementar). Grain size analysis was conducted at the British Geological Survey (Keyworth, UK) using laser diffraction (LS 13 320 Laser Diffraction Particle Size Analyzer, Beckman Coulter, UK), enabled with Polarization Intensity Differential Scattering which accounted for non-spherical, sub-micron particles as previously described^36,57^. Data analysis was conducted using Gradistat_v8^58^.

Radiocarbon analysis provides a model estimate of time since a carbon sample was in equilibrium with the atmosphere. All of these samples are bulk sedimentary OC, therefore, this time reflects the average age of the different carbon constituents and is related to the length of burial and lack of disturbance by active plant material. ^14^C samples were prepared to graphite at the NERC Radiocarbon Facility, East Kilbride and analysis was carried out at the SUERC AMS Laboratory, East Kilbride using a 5MV accelerator mass spectrometer, National Electrostatics Corporation, Wisconsin, US^59,60^ and the data reported in accordance with international practice^61^. Radiocarbon ages are reported as both ^14^C years before present (BP) and as calibrated (cal years BP). Radiocarbon calibration was undertaken using OxCal^62,63^ using the IntCal13 calibration curve^64^. Calibrated ages presented are the highest probabilities calculated from an analytical confidence of 2 sigmas. Where possible the calibrated ages are presented with a confidence of 95.4% but in some cases where a significantly narrower age range could be calculated from a moderately lower probability, this was accepted (see supplementary information Figures 7 and 8). δ^13^C of sedimentary OC is a bulk proxy commonly used to distinguish the source of different plant groups and marine and terrestrial inputs^19,30^. A sub-sample of CO_2_ (from the radiocarbon analysis) was used to measure δ^13^C using a dual-inlet mass spectrometer with a multiple ion beam collection facility (Thermo Fisher Delta V), in order to correct ^14^C data to −25‰ δ^13^C _VPDB_.

### Organic extraction, separation and analysis

Lipid biomarkers are used to analyse organic processes and sources of the sedimentary OC. Here, the term HMW refers to a carbon chain length of 21 to 35 for *n*-alkanes and 20 to 30 for *n*-alkanoic acids as the general regional pattern^11,12,14,16,17^. The concentration of HMW *n*-lipids (alkanes and alkanoic acids), both as a proportion of gram sediment and gram OC, is calculated to show the dominance of these compounds within the sediments and OC. The CPI, the proxy for thermal maturity, is calculated for all lipids and is the predominance of odd-over-even in the case of *n*-alkanes, and even-over-odd in the case of *n*-alkanoic acids^28,29^. CPI is calculated using the same method as van Dongen *et al*.^14^, and any value >2 is considered to be thermally immature. The average chain length (ACL) is calculated using the same method as van Dongen *et al*.^14^. The ratio of HMW *n*-alkanoic acids to Σ(HMW *n*-alkanes + HMW *n*-alkanoic) acids, e.g. the *n*-alkanoic acid to *n*-alkane ratio, is used to assess the extent of oxidation of OC^25,26^. This ratio is used because it expresses the end-members of organic oxidation and therefore the clearest separation of reduced and oxidised compounds. However, it is only suitable for those sediments which are immature, e.g. with HMW *n*-alkane CPI values  ≫ 2. This technique assumes that (i) any plant derived organic material will have a constant initial ratio of acids to alkanes and (ii) if different samples oxidise to different levels they will have different ratios of acids to alkanes^27^.

Organic analysis was conducted by extracting the total lipid extract using organic solvents and Soxhlet apparatus, followed by appropriate separation techniques based on earlier studies^11,14,16,26^. All analysis was conducted using Gas-Chromatography Mass Spectrometry (GCMS) using an Agilent 789 A GC interfaced to an Agilent 5975 C MSD. Full details are presented in the supplementary information.

### Data analysis and presentation

All statistical analysis was conducted using R^65^. Bulk sedimentary concentrations were kriged to produce geochemical maps using the package geoR^66^ (variograms shown in supplementary information). All statistical analyses were plotted using the R package ggplot2^67^.

## Electronic supplementary material

Supplementary Information
