# Supplementary material for: Biomarker-indicated extent of oxidation of plant-derived organic carbon (OC) in relation to geomorphology in an arsenic contaminated Holocene aquifer, Cambodia
Source: Sci Rep. 2017 Oct 12;7:13093. doi: 10.1038/s41598-017-13354-8 (PMC5638849; doi:10.1038/s41598-017-13354-8)
Supplement: Supplementary file 1 — Supplementary Information [file 41598_2017_13354_MOESM1_ESM.pdf]

Supplementary Information for:

Biomarker-indicated extent of oxidation of plant-derived organic carbon (OC) in relation to geomorphology in an arsenic contaminated Holocene aquifer, Cambodia

Daniel Magnone[1,2#], Laura A. Richards[1,2], David A. Polya[1,2], Charlotte Bryant[3], Merren Jones[1], Bart E. van Dongen[1,2*]

[1]School of Earth and Environmental Sciences, The University of Manchester, Williamson Building, Oxford Road, Manchester M13 9PL, United Kingdom

[2] Williamson Research Centre for Molecular Environmental Science, The University of Manchester, Williamson Building, Oxford Road, Manchester M13 9PL, United Kingdom

[3]NERC Radiocarbon Facility, Scottish Enterprise Technology Park, Rankine Avenue, East Kilbride, G75 OQF, UK

*Corresponding author: bart.vandongen@manchester.ac.uk

#Current address: School of Geography, University of Lincoln, Brayford Pool, Lincoln, Lincolnshire, LN6 7TS, UK.

Introduction

This supplementary information details methods, including quality assurance, used in this study for the determination in sediments of (i) TOC & TN; (ii) model radiocarbon age; (iii) $\delta^{13}C$; (iv) ordinary Kriging; (v) organic matter characterisation; and (vi) radiocarbon calibrations. Additionally is included data quality analysis for TOC, complete results for TOC, TN and grain-size analysis, variograms for TOC and grainsize, radiocarbon calibration plots and example chromatograms.

Detailed methodology

(i) Total organic carbon (TOC) was measured in the Faculty of Life Sciences, University of Manchester, using an elemental analyser (Vario EL Cube, Elementar). For data quality purposes total carbon (TC) and total nitrogen (TN) were also measured. TOC was measured using the capsule method [1]: approximately 20 mg of freeze-dried sample was accurately weighed into silver boats and placed on a hot plate. De-ionised water (10 µL, 18 MΩ) was added to the sample with the hot plate gradually heated up to 50 °C. Analytical reagent grade 5 % (w/w) hydrochloric acid (HCl, FisherBrand) was added to the silver boats in aliquots of 10 µL, 20 µL, 30 µL, 50 µL and 100 µL, not allowing the sample to dry out between each addition. After the addition of the 100 µL aqliuot of HCl the sample was left to dry out, rolled into a ball and analysed. Three CRMs of: low organic content (C 1.56 ± 0.06 % w/w, N 0.131 ± 0.009 % w/w) soil standard (cert. 242117, Elemental Microanalysis); medium organic content (C 3.19 ± 0.07 % w/w, N 0.27

± 0.02 % w/w) soil standard (cert. 115255, Elemental Microanalysis); and high organic content (C 7.17 ± 0.09 % w/w, N 0.57 ± 0.02 % w/w) soil standard (cert. 175032, Elemental Microanalysis) were used to check data quality. TN values from the sample capsule method were checked against pure TN values without acidification to check for any errors incurred. For unacidified TN samples were freeze dried, ground and approximately 20 mg of sediment was accurately weighed into tin sample boats and the boats were rolled into balls for analysis using the elemental analyser. TC, TOC and TN values were checked against an external laboratory for data quality (Elemental Lab, Okehampton, Devon, UK).

(ii) Samples for radiocarbon analysis and preparation were conducted at the NERC radiocarbon facility in East Kilbride. Samples were pre-treated to remove any carbonate from the sediments but minimising loss of organic carbon. After tests with sediments containing the highest amount of carbonate the following method was used. Samples were placed in pre-cleaned glass beakers, covered by glass fibre filter papers, placed into an evacuated glass container and exposed to concentrated hydrochloric acid fumes at 63 ± 2 °C to hydrolyze any carbonate in the sample. The samples were removed from the desiccator after 24 hours and stirred to ensure full exposure to acid fumes. They were fumigated for a further 24 hours. Graphite targets for ^{14}C analysis by AMS were prepared by quantitative recovery of carbon in sealed quartz tubes followed by cryogenic separation of CO_2 [2]. Aliquots of CO_2 were converted to an iron/graphite mix by iron/zinc reduction [3].

(iii) $\delta^{13}C$ was measured on sub-sample of CO_2 (from the radiocarbon analysis) using a dual-inlet mass spectrometer with a multiple ion beam collection facility (Thermo Fisher Delta V) in order to correct ^{14}C data to -25 ‰ $\delta^{13}C_{VPDB}$. The mass spectrometer was calibrated with international reference materials to a precision of $\pm$ 0.1 ‰. ^{14}C analysis was carried out at the SUERC AMS Laboratory, East Kilbride using a 5MV accelerator mass spectrometer, National Electrostatics Corporation, Wisconsin, US [4,5] and the data reported in accordance with international practice [6].

(iv) Ordinary kriging was undertaken in GeoR [7]. Kriging was undertaken on grain-size distribution using omnidirectional kriging the variogram for which is presented in Figure 5. For grainsize a spherical model was used to model distribution. Grainsize data proved to be strongly stratified horizontal to the surface. Due to the lower number of data points, geochemical kriging was undertaken using unidirectional variograms only. All

with the exception of sulphur were fitted with both spherical and exponential models and in all cases spherical models provided the best fit. Sulphur showed the poorest fit and this required a circular fitting model to provide a more accurate best fit. TOC Kriging was undertaken on the natural log of TOC values and then re-raised to the appropriate power for plotting to represent the non-logged values as this provided a better fit.

(v) Organic extraction, separation and analysis

Extraction of Total lipid extract (TLE) was undertaken in Soxhlet apparatus. Before extraction equipment (including Thimble filter) was cleaned by running a blank extraction for 24 hours. Approximately 30 to 50 g of freeze-dried and powdered sediment was placed within the Thimble filter in the Soxhlet. Unless stated otherwise all solvents were HPLC grade FisherBrand. Approximately 350 ml of a dichloromethane:methanol (DCM:MeOH, 2:1 v/v) mixture was placed in a round-bottomed flask at the base of the Soxhlet with anti-bumping granules, and heated to 40 °C with a heating mantle to induce boiling for 24 hours. Solvents were evaporated using rotary evaporation, the TLE transferred to a vial, blown dry under a stream of nitrogen and frozen before further separation.

Separation of a TLE was undertaken on an aliquot of the total sample (about 30 % of the TLE depending on recovery) using bond elute chromatography with glass solid phase extraction (SPE) columns (Biotage Isolute NH_2 500 mg / 6 ml, 470-0050-L). SPE columns were cleaned by running 12 ml of MeOH, 12 ml of isopropanol:DCM (iPA:DCM, 2:1 v/v) and finally 12 ml of 4% glacial acetic acid (Fluka) in diethyl ether through the column; at no point were the columns permitted to run dry. The aliquot was dissolved in iPA:DCM (2:1 v/v), two internal standards, 540 ng of tetracosane-d50 (Fluka) and 5400 ng of 2-hexadecanol (Fluka), were added and transferred to toc column. The aliquot was separated by eluting with 12 ml of iPA:DCM (neutral fraction; F1), 12 ml of 4% glacial acetic acid in diethyl ether (acid fraction; F2) and 12 ml MeOH (remaining lipids;F3). F1 and F3 were dried using rotary evaporation whilst F2 was dried under a stream of nitrogen. All fractions were transferred to vials using DCM, dried under nitrogen and kept frozen until further analysis (whilst F3 was collected it was not used during this study).

Fraction F1 was dissolved in hexane/DCM (9:1 v/v) and passed through a column packed with activated Al_2O_3. This was eluted into an apolar fraction with 3 ml hexane/DCM, and, a polar fraction with DCM/MeOH (1:1 v/v, 3 ml). The apolar fraction was dried under a

flow of nitrogen, dissolved in hexane and analysed using gas chromatography-mass spectrometry (GC-MS, see settings below). The polar fraction was dried using nitrogen and silylated by adding 25 µL bis(trimethylsilyl)trifluoroacetamide (BSTFA), heating at 70 ˚C for 1 hour in a closed vial, left to cool, dried completely under nitrogen and dissolved in hexane for analysis by GCMS. 540 ng of tetracosane-d_{50} was added to F2 before methylation by adding 100 µL of BF_3/MeOH, heating at 70 ˚C for 1 hour in a closed vial. After cooling 1 mL of (extracted) bi-distilled water was added and the water layer was extracted with DCM. The remaining DCM layer was passed through Na_2SO_4 to remove any water, dried under nitrogen and silylated using the same method as the polar fraction before analysis by GCMS. These techniques are consistent with earlier studies in the region [8–11].

GCMS was performed using an Agilent 789A GC interfaced to an Agilent 5975C MSD mass spectrometer operated with electron ionisation at 40 eV and scanning from *m/z* 50 to 650 for all samples and also simultaneous SIM mode of *m/z* 57 and 66, and *m/z* 74 and 100 for alkanes and alkanoic acids, respectively. The GC was equipped with an Agilent 7683B auto sampler and a programmable temperature variable (PTV) injector. The samples were injected using a pulsed split-less injection (1 µl inlet pressure of 25 psi for 0.25 mins) and separated on an Zebron ZB-5MS capillary column (5%-Phenyl Arylene, 95% Dimethyl Polysiloxane: length: 30 m, ID 250 µm, film thickness 0.25 µm). The samples were run at constant flow (1 ml/min) with He as a carrier gas. The heated interface temperature was set to 280 ^{o}C, with the main oven temperature at 230 ^{o}C and the MS quadrupole at a temperature of 150 ^{o}C. The samples were injected at 50 ^{o}C and the oven was programmed to 320 ^{o}C at 7 ^{o}C/min whilst it was held constant for 10 mins. The compounds were identified by comparison of retention times of mass spectra with those present in the literature. Quantitative data were obtained by comparing the individual peaks to that of the known internal standard added.

(vi) Radiocarbon Calibration Methods

Within this manuscript ^{14}C years BP refers to the radiocarbon age without calibration and cal years BP refers to the calibrated dates. Calibration of radiocarbon years to calendar age ranges was conducted using OxCal version 4.2 [12] and IntCal13 calibration curve [13].

This section provides the code used for all calibrations presented in this study and provides the OxCal plots for the data collected by this study but not the earlier studies

[8,10,14]. For all samples except LR05, simple single radiocarbon calibration was undertaken (Figure 1 and Figure 2). For LR05 a P_Sequence was also used (Figure 3) this applies Bayesian logic to a depth profile assume that all samples age with depth this provides a more accurate calibration where this technique is suitable – LR05 was the only site which fit the criteria.

```
Plot()
 {
  R_Date("LR01-6m-SED", 1588, 37);
  R_Date("LR01-30m-SED", 3705, 36);
  R_Date("LR10-6m-SED", 2555, 35);
  R_Date("LR10-27m-SED", 3393, 37);
  R_Date("LR14-6m-SED", 6295, 39);
  R_Date("LR14-30m-SED", 9450, 41);
  R_Date("LR09-6m-SED", 1430, 35);
  R_Date("LR09-21m-SED", 3911, 35);
  R_Date("LR09-30m-SED", 3597, 35);
  R_Date("LR09-39m-SED", 8002, 40);
  R_Date("LR09-45m-SED", 2930, 35);
 };
```

Figure 1 OxCal code for radiocarbon calibration of the sedimentary data collected for this study.

```
Plot()
 {
  R_Date("SR-13", 7759, 51);
  R_Date("SR-19", 7732, 51);
  R_Date("DS-0", 6216, 44);
  R_Date("DS-15", 8177, 54);
  R_Date("DS-23", 7930, 52);
  R_Date("DS-27", 9040, 61);
  R_Date("DS-40", 5370, 41);
```

```
  R_Date("DS-54", 5293, 41);
  R_Date("DS-60", 8241 , 41);
  R_Date("DS-70", 4937, 41);
  R_Date("SY-9", 1532, 31);
  R_Date("SY-28", 4218, 38);
  R_Date("KS-4.07", 700, 40);
  R_Date("KS-7.08", 6250, 40);
  R_Date("KS-7.9", 6620, 40);
  R_Date("KS-8.33", 6470, 40);
  R_Date("KS-9.08", 7130, 40);
  R_Date("KS-9.6", 7030, 40);
  R_Date("KS-10.48", 7150, 40);
  R_Date("KS-12.27", 6550, 40);
  R_Date("KS-12.65", 6760, 40);
  R_Date("KS-28.10", 8180, 40);
 };
```

Figure 2 OxCal code for radiocarbon calibration of the sedimentary data collected by previous studies [8,10,14].

```
Plot()
 {
  Outlier_Model("General",T(5),U(0,4),"t");
  P_Sequence("",100,4,U(-2,2))
  {
   Boundary();
   R_Date("LR05-45m",12011,45)
   {
    Outlier(0.05);
    z=45;
   };
   R_Date("LR05-30m",9432,40)
```

```
    {
     Outlier(0.05);
     z=30;
    };
    R_Date("LR05-15m",3363,35)
    {
     Outlier(0.05);
     z=15;
    };
    R_Date("LR05-9m",1923,35)
    {
     Outlier(0.05);
     z=9;
    };
    R_Date("LR05-6m",1683,35)
    {
     Outlier(0.05);
     z=6;
    };
    Boundary();
   };
  };
```

Figure 3 OxCal code for radiocarbon calibration of the sedimentary data from LR05 using P_Sequence to provide baysian approach to calibration for a more accurate date.

TOC data quality results

To check data quality of the TOC analysis the residuals of TOC measured at the external laboratory (x) versus TOC measured at Manchester (y) were analysed for a line of y = x. These showed a systematic increase in residual from >-0.05 when TOC was <1 % as measured at Manchester to about -0.12. This means that where samples where TOC was >0.2 % the residual percentage was <10%. For samples with TOC value of <0.05 % the residual percentage extremely high (Figure 4).

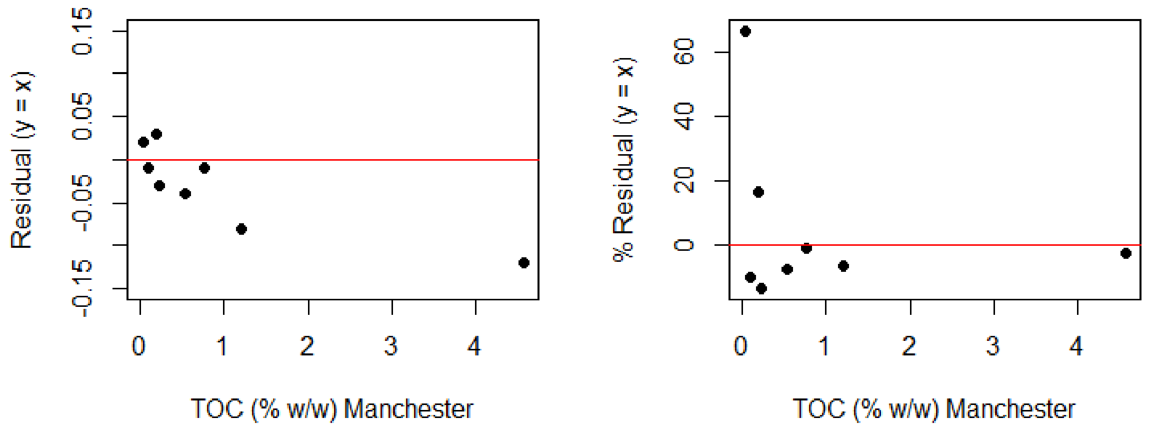

Figure 4 the residuals of TOC measured at the external laboratory (x) versus TOC measured at Manchester (y) were analysed for a line of y = x and corresponding percentages.

Bulk analysis results

This section provides the bulk sedimentary values and confidences referred to in the main body of the text (Table 1).

Table 1 Bulk concentrations for aquifer sediments in Kandal Province, Cambodia.

**Sample** [a]	**TOC % (wt/wt)** [b]	**TOC St. Dev.** [c]	**TN % (wt/wt)** [d]	**TN St. Dev.** [e]	**C/N** [f]	**Grain size mean (µm)** [g]	**Grain size sorting (µm)** [h]	**Grain size skew** [i]
LR01-3	0.32	0.003	0.06	0.002	5.84	9.005	2.904	-0.060
LR01-6	1.20	0.041	0.09	0.003	12.83	40.04	3.756	-0.527
LR01-9	0.30	0.010	0.02	0.002	12.52	65.79	3.684	-0.464
LR01-12	n.d.	n.d.	n.d.	n.d.	n.d.	99.95	4.211	-0.647
LR01-15	0.10	0.008	0.01	0.001	7.29	118.0	3.198	-0.470
LR01-18	n.d.	n.d.	n.d.	n.d.	n.d.	102.2	5.771	-0.639
LR01-21	0.08	0.006	0.01	0.002	5.57	231.8	2.203	-0.180
LR01-24	n.d.	n.d.	n.d.	n.d.	n.d.	153.1	4.543	-0.483
LR01-27	n.d.	n.d.	n.d.	n.d.	n.d.	93.25	7.920	-0.369
LR01-30	0.07	0.031	0.01	0.001	6.90	59.99	7.416	-0.390
LR01-36	0.18	0.036	0.02	0.001	7.86	95.74	4.836	-0.676
LR01-39	0.15	0.013	0.02	0.002	8.58	n.d.	n.d.	n.d.
LR02-3	0.27	0.024	0.03	0.004	9.43	59.64	3.611	-0.533
LR02-6	n.d.	n.d.	n.d.	n.d.	n.d.	87.10	3.416	-0.529
LR02-9	0.44	0.016	0.03	0.003	13.44	80.31	4.100	-0.617
LR02-15	0.22	0.039	0.03	0.006	7.98	114.1	3.945	-0.578
LR02-21	0.24	0.021	0.02	0.003	15.79	927.3	1.784	-0.375

LR02-27	0.07	0.017	0.01	0.005	8.01	1073.9	1.670	-0.339
LR02-30	0.96	0.028	0.26	0.300	3.66	n.d.	n.d.	n.d.
LR03-3	0.44	0.030	0.06	0.001	7.68	54.54	5.109	-0.556
LR03-6	0.30	0.010	0.03	0.006	8.92	151.5	2.739	-0.681
LR03-9	0.68	0.026	0.05	0.002	12.55	90.65	3.884	-0.673
LR03-15	0.04	0.049	0.03	0.003	1.51	123.3	3.861	-0.625
LR03-21	0.62	0.012	0.06	0.001	11.12	169.4	6.430	-0.523
LR03-30	n.d.	n.d.	n.d.	n.d.	n.d.	14.37	3.155	-0.103
LR04-3	0.43	0.018	0.05	0.004	9.15	12.11	3.624	0.037
LR04-6	n.d.	n.d.	n.d.	n.d.	n.d.	112.4	2.935	-0.513
LR04-9	0.25	0.029	0.02	0.002	12.18	92.69	3.826	-0.690
LR04-12	0.21	0.020	0.02	0.005	10.17	85.13	2.062	-0.167
LR04-18	0.17	0.022	0.02	0.006	9.25	n.d.	n.d.	n.d.
LR04-21	0.16	0.019	0.02	0.001	10.78	240.5	1.690	0.005
LR04-30	0.74	0.060	0.05	0.002	13.89	20.70	3.533	-0.132
LR05-3	0.18	0.019	0.03	0.000	6.07	34.83	3.770	-0.486
LR05-6	0.77	0.012	0.07	0.001	10.71	19.32	3.517	-0.137
LR05-9	0.37	0.025	0.04	0.002	10.14	59.19	3.719	-0.482
LR05-12	n.d.	n.d.	n.d.	n.d.	n.d.	90.87	3.899	-0.637
LR05-15	0.10	0.012	0.01	0.001	7.37	198.4	2.423	-0.599
LR05-21	0.06	0.034	0.01	0.004	9.31	124.6	3.328	-0.426
LR05-24	n.d.	n.d.	n.d.	n.d.	n.d.	n.d.	n.d.	n.d.
LR05-27	n.d.	n.d.	n.d.	n.d.	n.d.	594.7	1.607	-0.107
LR05-30	1.19	0.040	0.11	0.002	10.59	9.254	2.717	-0.039
LR05-36	0.98	0.008	0.10	0.003	9.33	9.552	2.696	-0.091
LR05-39	0.93	0.008	0.14	0.009	6.53	9.950	3.127	-0.036
LR05-45	0.35	0.011	0.07	0.004	4.78	5.637	3.281	-0.230
LR16-15	0.41	0.082	0.05	0.004	9.19	n.d.	n.d.	n.d.
LR16-30	0.90	0.017	0.10	0.007	9.25	n.d.	n.d.	n.d.
LR06-3	0.33	0.026	0.04	0.001	8.01	n.d.	n.d.	n.d.
LR06-9	0.36	0.032	0.03	0.004	11.09	n.d.	n.d.	n.d.
LR06-15	0.10	0.014	0.02	0.008	5.34	n.d.	n.d.	n.d.
LR06-27	0.11	0.012	0.01	0.006	10.07	n.d.	n.d.	n.d.
LR07-3	0.26	0.027	0.03	0.001	8.07	18.18	3.733	-0.085
LR07-6	0.51	0.025	0.05	0.005	9.50	50.77	3.426	-0.554
LR07-9	0.50	0.018	0.05	0.002	10.39	47.14	3.798	-0.520
LR07-15	0.15	0.024	0.02	0.007	7.30	123.2	2.665	-0.559
LR07-21	0.19	0.024	0.02	0.002	11.47	112.1	4.133	-0.688
LR07-30	0.61	0.085	0.04	0.008	16.50	44.74	7.148	-0.261
LR08-3	0.34	0.015	0.04	0.001	8.32	17.58	3.785	-0.110
LR08-6	n.d.	n.d.	n.d.	n.d.	n.d.	35.67	3.942	-0.406
LR08-9	0.52	0.013	0.04	0.001	11.86	60.96	3.456	-0.643

LR08-15	0.30	0.019	0.05	0.006	5.88	45.40	4.623	-0.428
LR08-21	0.33	0.032	0.04	0.003	9.04	68.96	4.137	-0.589
LR08-30	0.12	0.019	0.02	0.007	6.43	170.9	3.324	-0.683
LR09-3	0.17	0.015	0.04	0.003	4.59	13.39	4.486	-0.166
LR09-6	1.27	0.074	0.09	0.006	13.57	22.05	3.571	-0.164
LR09-9	0.54	0.051	0.05	0.002	10.59	44.45	3.457	-0.534
LR09-12	n.d.	n.d.	n.d.	n.d.	n.d.	34.67	3.947	-0.325
LR09-15	0.15	0.037	0.03	0.007	5.75	83.99	2.462	-0.558
LR09-18	n.d.	n.d.	n.d.	n.d.	n.d.	14.90	3.730	0.044
LR09-21	0.14	0.044	0.02	0.004	7.01	87.75	3.122	-0.612
LR09-24	n.d.	n.d.	n.d.	n.d.	n.d.	168.8	2.281	-0.544
LR09-27	n.d.	n.d.	n.d.	n.d.	n.d.	110.6	4.729	-0.430
LR09-30	0.01	0.005	0.00	0.001	1.78	548.3	2.077	-0.202
LR09-39	0.04	0.013	0.02	0.006	2.23	46.80	6.803	-0.091
LR09-45	0.06	0.026	0.02	0.012	3.83	299.1	2.617	-0.056
LR10-3	0.22	0.038	0.03	0.005	6.38	38.50	5.078	-0.347
LR10-6	0.17	0.015	0.03	0.019	5.58	81.35	4.225	-0.611
LR10-9	0.19	0.013	0.03	0.006	6.53	92.03	3.961	-0.615
LR10-12	0.42	0.031	0.03	0.002	13.66	86.30	3.617	-0.536
LR10-15	0.18	0.014	0.02	0.007	8.18	244.5	1.957	-0.463
LR10-21	0.09	0.019	0.02	0.007	4.50	178.2	2.651	-0.606
LR10-24	0.35	0.044	0.03	0.002	11.47	247.1	1.732	-0.361
LR10-27	0.17	0.036	0.02	0.003	9.37	n.d.	n.d.	n.d.
LR10-30	0.10	0.015	0.02	0.007	6.38	243.6	2.181	-0.424
LR14-3	0.31	0.028	0.06	0.002	5.20	7.943	3.207	-0.040
LR14-6	0.15	0.022	0.05	0.003	3.23	6.232	3.694	-0.195
LR14-9	1.97	0.011	0.12	0.002	15.81	11.82	3.038	-0.040
LR14-15	1.25	0.072	0.14	0.006	8.73	40.47	3.507	-0.492
LR14-21	0.26	0.004	0.04	0.001	6.99	57.23	3.153	-0.618
LR14-30	0.47	0.104	0.05	0.008	9.73	34.71	4.177	-0.346

[a] Samples labelled LRXX-## where LRXX refers to the location (see Figure 1 main text) and ## refers to the depth.

[b] Total organic carbon concentration % (w/w).

[c] Standard deviation of total organic carbon % (w/w) (n =3).

[d] Total nitrogen concentration % (w/w).

[e] Standard deviation of total nitrogen % (w/w) (n =3).

[f] Carbon to nitrogen ratio.

[g] Mean Folk and Ward grain size reported µm.

[h] Mean Folk and Ward grain sorting to one standard deviation reported µm.

[i] Folk and Ward grainsize skew .

n.d. no data.

Variogram results

Below are the two variograms for the T-Sand cross-sections used in this paper.

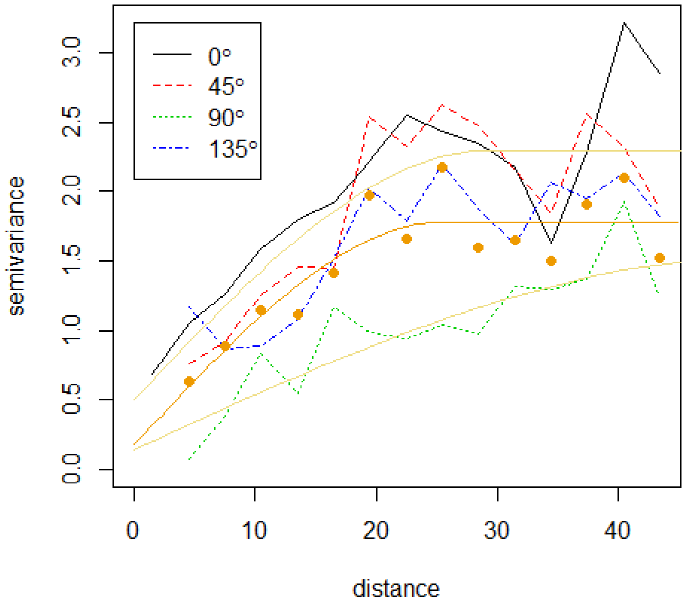

Figure 5 Omnidirectional variogram of grainsize data for study shown with lines (line styles and colours representing different directions) and uni-directional kriging shown with points. Spherical models shown with smooth fitted orange lines.

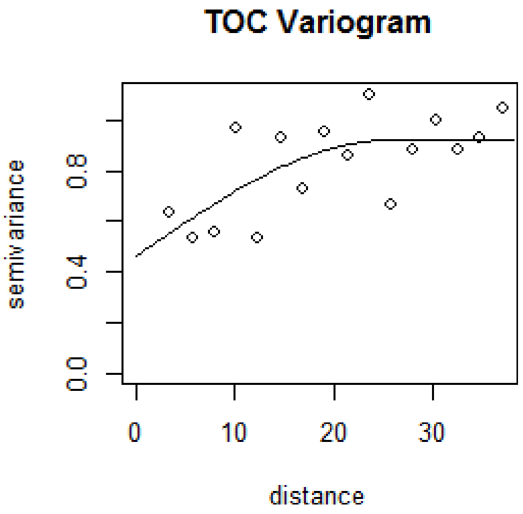

Figure 6 Variograms of TOC data plotted for this study with corresponding models.

Radiocarbon calibration results

The following figures show the OxCal plots for radiocarbon calibration. Where possible all samples were reported to a 95.4% probability confidence, however, a small number of sites (LR01-30, LR10-27, LR09-30 and LR14-30) were reported to lower probabilities because where a lower probability outlier increased the calibration ages significantly (Figure 7).

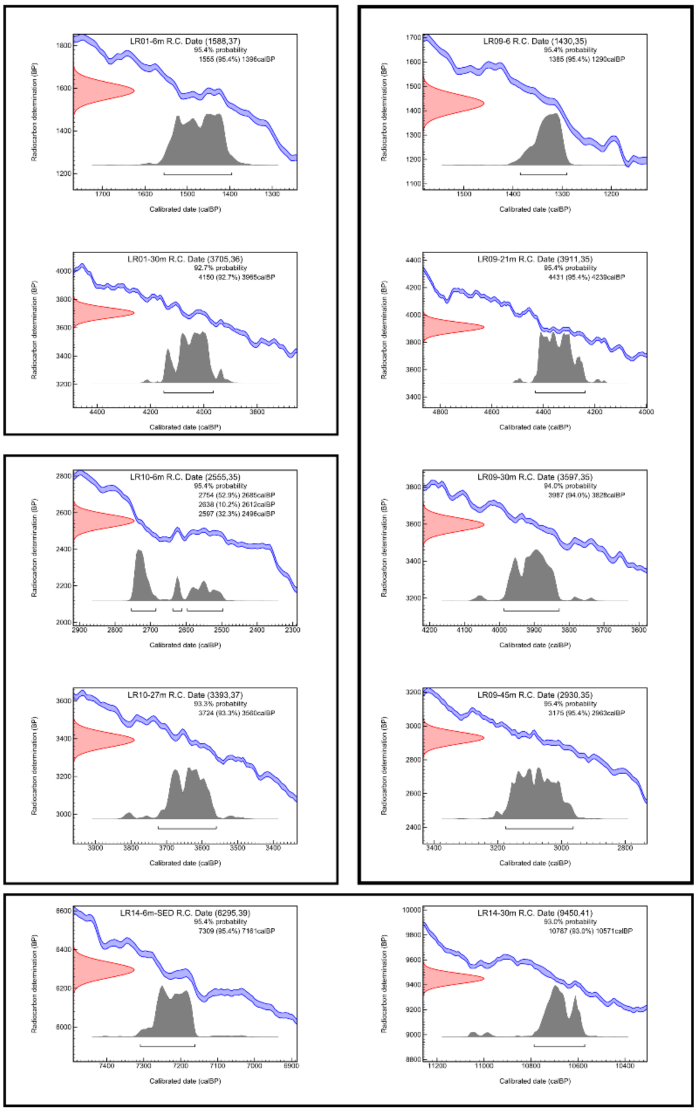

Figure 7 OxCal plots for radiocarbon calibration by this study grouped by site. Red distribution is measured data, grey is the probability distribution of the calibrated date and the blue line is the calibration curve.

At LR05 the Bayesian approach had high levels of agreement with the simple calibration approach in all samples. Where possible all samples were reported to a 95.4% probability confidence, however, at LR05-6, LR05-15 and LR05-30 lower probabilities were used to provide a more constrained calibration (Figure 8).

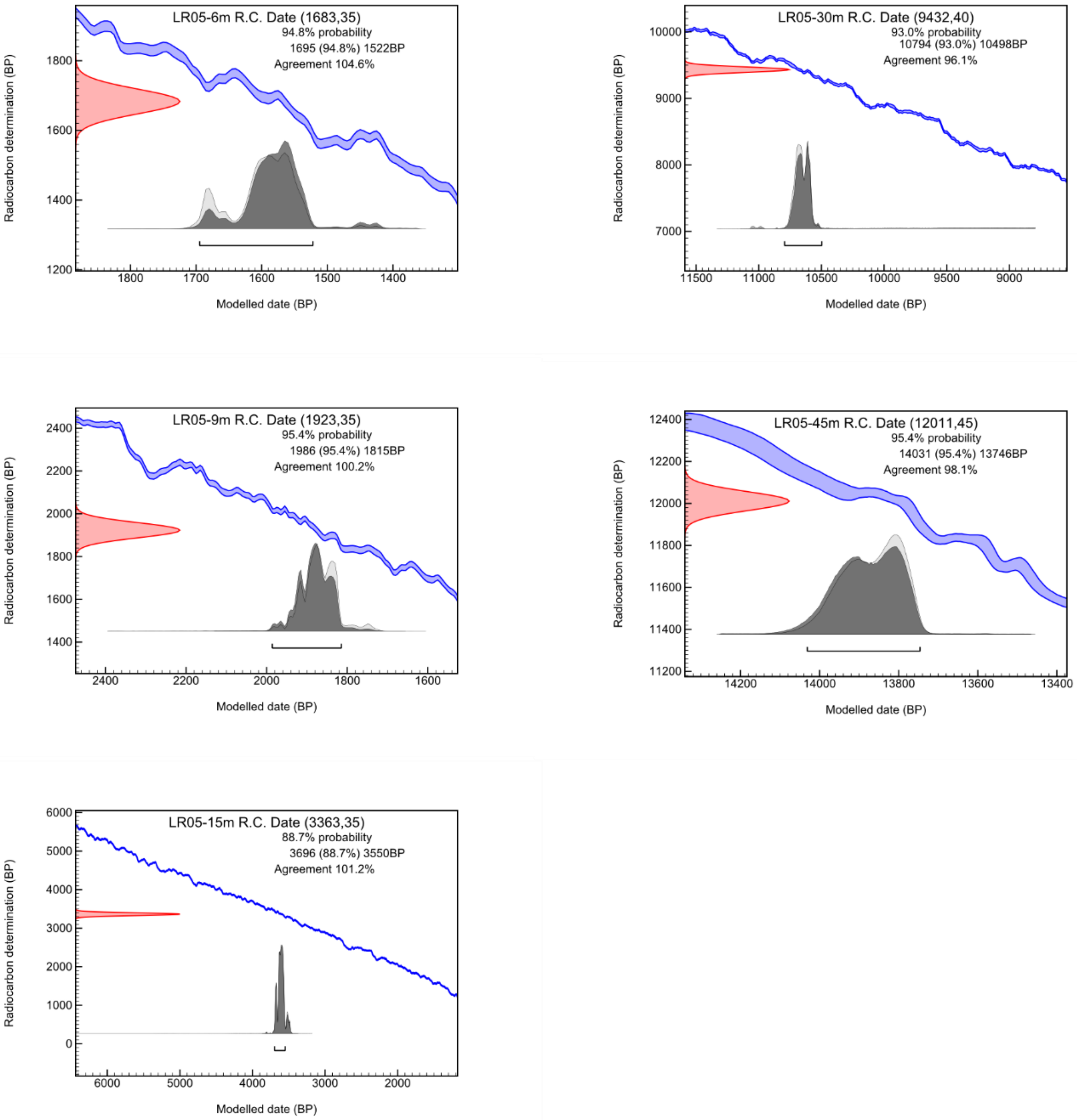

Figure 8 OxCal plots showing conventional simple calibration in light grey and Bayesian calibration in darker grey. Red distribution is measured data and the blue line is the calibration curve.

Chromatography Results

For completeness a selection of representative scanned (not SIM) chromatograms are shown in Figure 9. The *n*-alkanes from LR05-6m represent the immature (i.e. high CPI) *n*-alkanes whilst the *n*-alkanes from LR05-45m show the immature (*i.e.* low CPI) *n*-alkanes. LR09-6m shows typical *n*-alkanoic acids and LR14-9m shows typical *n*-alcohols.

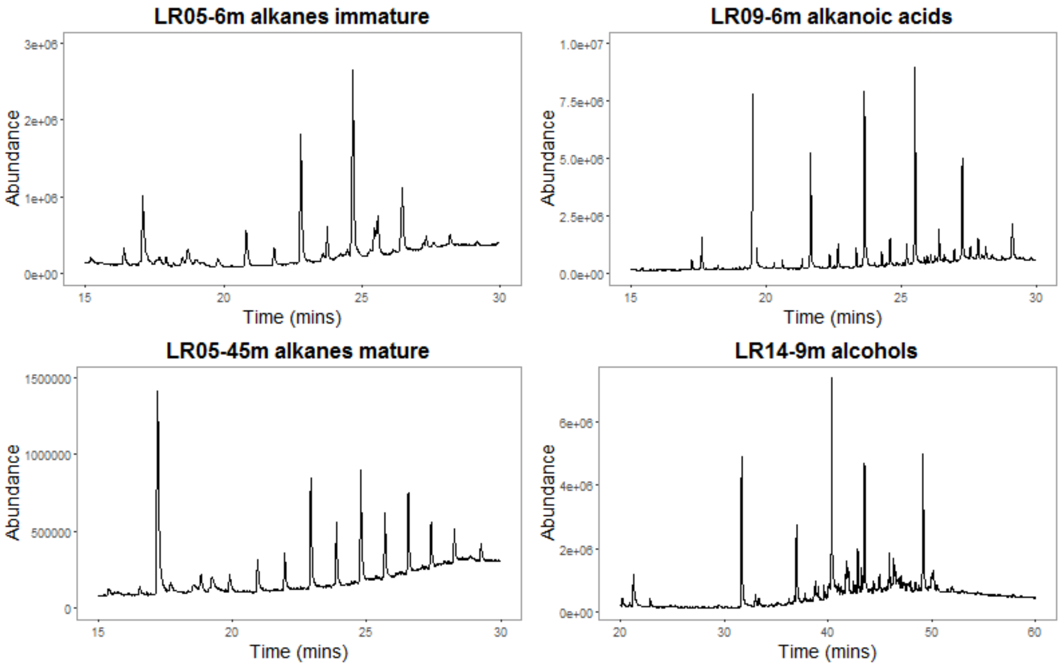

Figure 9 Representative chromatograms for different compounds and maturities for data collected by this study. The internal standard in the alkane phases occurs at about 17 mins and at 19 mins for the alkanoic acids and is not shown in the alcohols.

References

1. Brodie, C. R. *et al.* Evidence for bias in C and N concentrations and δ13C composition of terrestrial and aquatic organic materials due to pre-analysis acid preparation methods. *Chem. Geol.* **19,** 65–67 (2011).

2. Boutton, T. W. *et al.* Comparison of quartz and pyrex tubes for combustion of organic samples for stable carbon isotope analysis. *Anal. Chem.* **55,** 1832–1833 (1983).

3. Slota, P., Jull, A. J. T., Linick, T. W. & Toolin, L. J. Preparation of small samples for 14C accelerator targets by catalytic reduction of CO. *Radiocarbon* **29,** 303–306 (1987).

4. Xu, S., Anderson, R., Bryant, C. & Cook, G. Capabilities of the New SUERC 5MV AMS Facility for 14C Dating. *Radiocarbon* **46,** 59–64 (2004).

5. Freeman, S. P. H. T., Dougans, A., McHargue, L., Wilcken, K. M. & Xu, S. Performance of the new single stage accelerator mass spectrometer at the SUERC. *Nucl. Instruments Methods Phys. Res. Sect. B Beam Interact. with Mater. Atoms* **266,** 2225–2228 (2008).

6. Stuiver, M. & Polach, H. A. Reporting of C-14 data–discussion. *Radiocarbon* **19,** 355–363 (1977).

7. Ribeiro, P. J. J. & Diggle, P. J. geoR: Analysis of Geostatistical Data. R package version 1.7-5.1. (2015).

8. Rowland, H. A. L. *et al.* The control of organic matter on microbially-mediated iron reduction and arsenic release in shallow alluvial aquifers. *Geobiology* **5,** 281–292 (2007).

9. Rowland, H. A. L., Polya, D. A., Lloyd, J. R. & Pancost, R. D. Characterisation of organic matter in a shallow, reducing, arsenic-rich aquifer, West Bengal. *Org. Geochem.* **37,** 1101–1114 (2006).

10. van Dongen, B. E. *et al.* Hopane, sterane and n-alkane distributions in shallow sediments hosting high arsenic groundwaters in Cambodia. *Appl. Geochemistry* **23,** 3047–3058 (2008).

11. Al Lawati, W. M. *et al.* Characterisation of organic matter and microbial communities in contrasting arsenic-rich Holocene and arsenic-poor Pleistocene aquifers, Red River Delta, Vietnam. *Appl. Geochemistry* **27,** 315–325 (2012).

12. Bronk Ramsey, C. & Lee, S. Recent and Planned Developments of the Program OxCal. *Radiocarbon* **55,** 720–730 (2013).

13. Reimer, P. J. *et al.* Intcal13 and Marine13 Radiocarbon Age Calibration Curves 0–50,000 Years Cal Bp. *Radiocarbon* **55,** 1869–1887 (2013).

14. Tamura, T. *et al.* Depositional facies and radiocarbon ages of a drill core from the Mekong River lowland near Phnom Penh, Cambodia: Evidence for tidal sedimentation at the time of Holocene maximum flooding. *J. Asian Earth Sci.* **29,** 585–592 (2007).
